# Navigating Cervical Cancer Risk in a Patient With Classic Congenital Adrenal Hyperplasia (CAH): A Case Report

**DOI:** 10.7759/cureus.89331

**Published:** 2025-08-04

**Authors:** Anjiya Aswani, Elaina Butt, Oluwaseyi Akinooye, Chonnikarn Chokapirat, Oransaye Osaretin

**Affiliations:** 1 Obstetrics and Gynecology, Ross University School of Medicine, Bridgetown, USA; 2 Family Medicine, Mount Sinai Hospital, Chicago, USA; 3 Obstetrics and Gynecology, Mount Sinai Hospital, Chicago, USA

**Keywords:** cancer cervical, cervical intraepithelial neoplasia (cin iii), congenital adrenal hyperplasia, gynecologic oncology, hiv-positive, hyperandrogenism, vesicovaginal fistula

## Abstract

Cervical cancer is rarely reported in patients with congenital adrenal hyperplasia (CAH), a condition that may alter risk through hormonal and anatomical factors. When combined with human immunodeficiency virus (HIV), the risk of progression from cervical intraepithelial neoplasia (CIN) to invasive carcinoma may be amplified, yet remains underreported. We report the case of a 57-year-old woman with a history of classic CAH, HIV, cervical intraepithelial neoplasia grade III (CIN III), hypertension, and asthma who presented with abdominal pain and pneumaturia. Imaging revealed air within the vaginal cuff and endometrial canal, raising concern for a fistula. Further evaluation confirmed invasive cervical cancer complicated by vesicovaginal fistula formation. This case highlights the complex interplay between immunosuppression and hormonal dysregulation in cervical cancer pathogenesis. Despite known risk factors, the diagnosis was delayed due to atypical presentation and poor follow-up. Fistula formation added further morbidity and required coordinated multidisciplinary care. In patients with CAH and HIV, cervical cancer may present atypically and be complicated by fistula development. Clinicians should maintain a high index of suspicion and pursue early, coordinated evaluation in patients with overlapping immunologic and endocrine comorbidities.

## Introduction

Cervical cancer remains a significant cause of morbidity and mortality worldwide, particularly among immunocompromised populations such as patients living with human immunodeficiency virus (HIV) [1]. Congenital adrenal hyperplasia (CAH) is a rare group of inherited disorders affecting adrenal steroidogenesis, with limited literature on its potential impact on gynecologic health [2]. Patients with CAH may experience hormonal imbalances that could theoretically influence cervical epithelium homeostasis. 

This case report describes a 57-year-old woman with a complex medical history, including hypertension, HIV, CAH, and cervical intraepithelial neoplasia grade III (CIN III), who presented with abdominal pain, vaginal bleeding, and malodorous discharge. Imaging revealed concerning findings for a rectovaginal fistula, an uncommon complication associated with advanced cervical pathology. This report highlights the diagnostic challenges and management considerations in a patient with overlapping immunologic and endocrine disorders, emphasizing the need for multidisciplinary care and vigilant follow-up in such complex cases.

## Case presentation

A 57-year-old woman with a past medical history of hypertension, HIV, CAH, and CIN III presented to the emergency department with abdominal pain.

Approximately five days before admission, she developed intermittent, sharp, non-radiating lower abdominal pain localized predominantly to the left lower quadrant. This was accompanied by nausea without vomiting and diarrhea. She reported a small amount of bright red blood mixed with stool during initial diarrheal episodes, followed by daily loose bowel movements without visible blood. Since the onset of diarrhea, she experienced intermittent vaginal bleeding without clots.

The patient endorsed a known history of cervical dysplasia but admitted to noncompliance with gynecologic follow-up. She denied appetite changes, unintended weight loss, fatigue, or other constitutional symptoms. She reported intermittent fevers, chills, and urinary frequency but denied urinary urgency, hematuria, or pyuria.

On presentation, the patient was afebrile and hemodynamically stable. Laboratory investigations (Table 1) were notable for acute kidney injury with a creatinine of 3.00 mg/dL and leukocytosis with a white blood cell count of 13.90 × 10^3^/mcL. Urinalysis was positive for leukocyte esterase. Influenza and COVID-19 tests were negative.

**Table 1:** Laboratory values on day one of admission. BUN: blood urea nitrogen; GFR: glomerular filtration rate; WBC: white blood cell.

Component	Value	Normal range
Sodium	142 mEq/L	133-144 mEq/L
Potassium	4.2 mEq/L	3.5-5.2 mEq/L
Anion gap	16.4 mEq/L	6-15 mEq/L
BUN	49 mg/dL	7-25 mg/dL
Creatinine, blood	3.00 mg/dL	0.6-1.2 mg/dL
GFR	18	≥60
WBC	13.90 x 10^3^/mcL	4.00-11.00 x 10^3^/mcL
Hemoglobin	13.1 g/dL	12.0-15.3 g/dL
Urine WBC esterase	500 Leu/uL	Negative
Urine WBC	>200	0-4/hpf

Gynecology was consulted and requested a pelvic ultrasound, which showed a uterus measuring 7.7 × 4.4 × 3.4 cm with an endometrial thickness of 3.7 mm and echogenic foci within the endometrial cavity; ovaries were not visualized.

**Figure 1:**
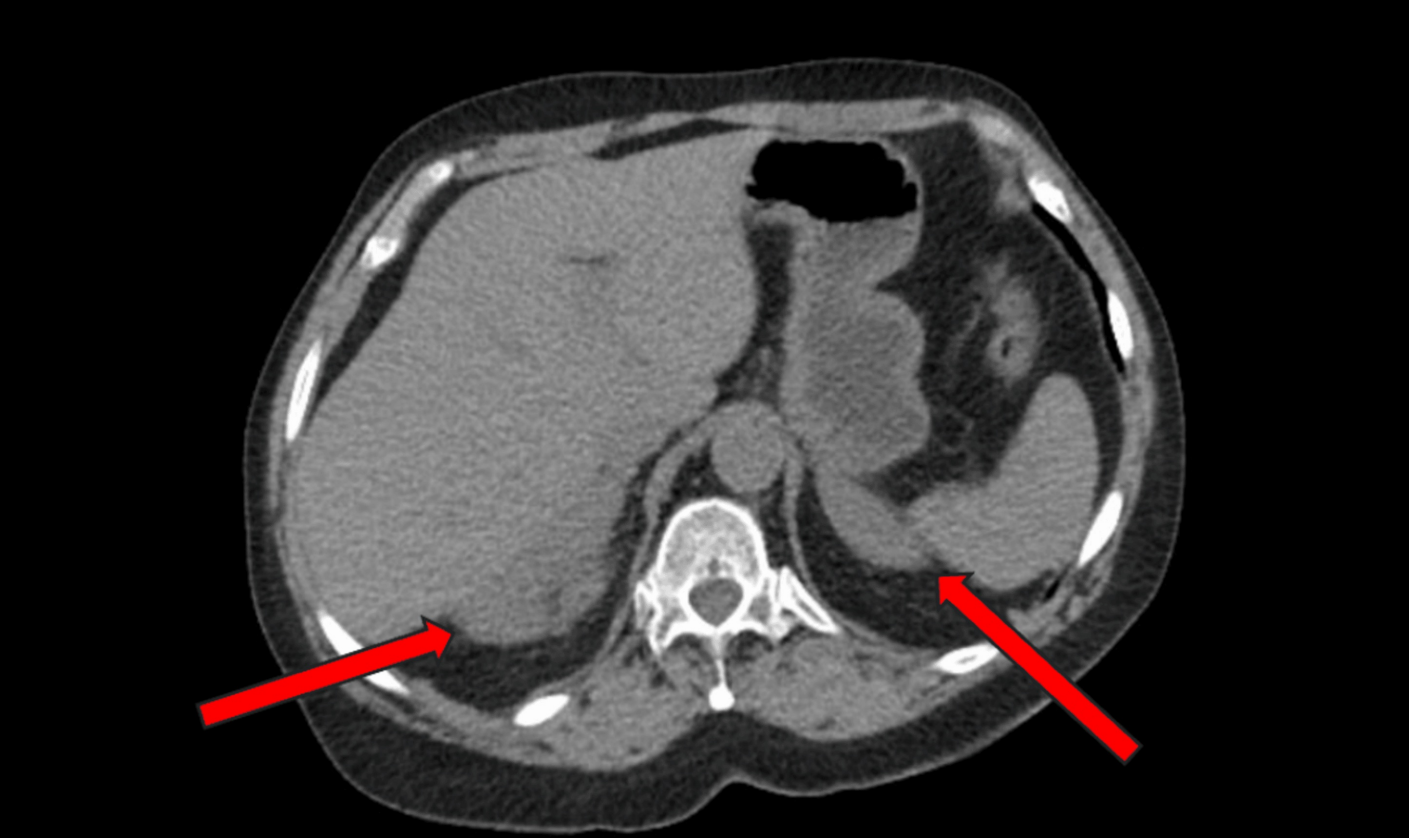
CT abdomen and pelvis demonstrating bilateral adrenal gland enlargement (indicated by red arrows). The adrenal glands appear diffusely enlarged without discrete masses, consistent with adrenal hyperplasia.

**Figure 2:**
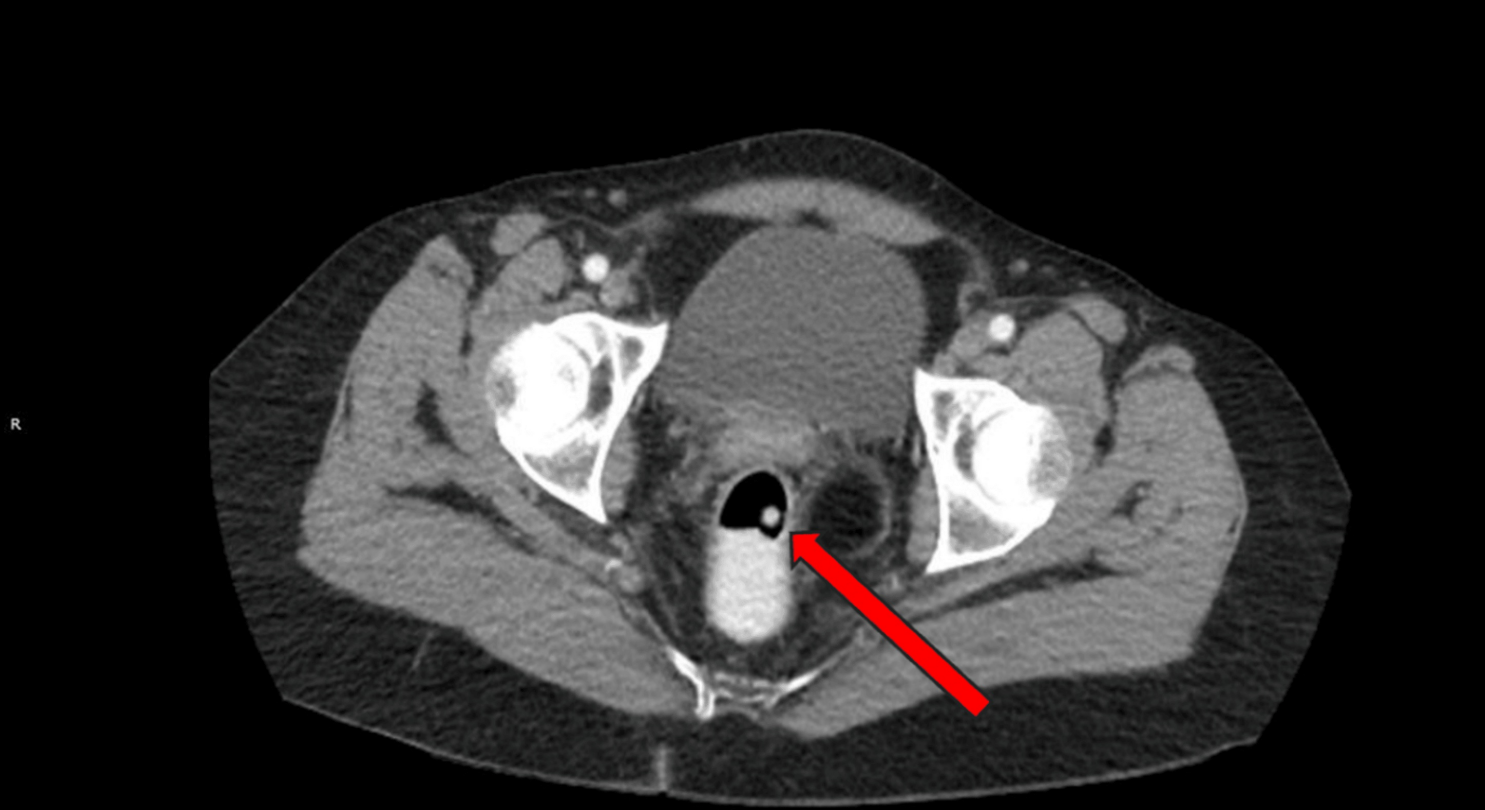
CT image of the pelvis demonstrating abnormal intraluminal air within the vaginal canal (indicated by red arrow).

Computed tomography scan of the abdomen and pelvis on admission revealed bilateral adrenal gland enlargement (Figure 1), air within the vaginal cuff (Figure 2) and endometrial canal, left fallopian tube thickening, and right renal punctate nephrolithiasis. 

The patient was treated with intravenous fluids, morphine for pain, and ceftriaxone for suspected urinary tract infection (UTI). She was admitted with a diagnosis of UTI and acute kidney injury (AKI), meeting sepsis criteria. Gastroenterology and gynecology were consulted for evaluation of a possible fistula and CIN III. After five days, she was discharged with outpatient follow-up arranged for colonoscopy and gynecologic assessment.

## Discussion

This case highlights the diagnostic and therapeutic complexity of cervical cancer in a patient with coexisting congenital adrenal hyperplasia (CAH), human immunodeficiency virus (HIV) infection, and cervical intraepithelial neoplasia grade III (CIN III). Each of these conditions independently presents unique challenges to gynecologic care, and their intersection in a single patient underscores the need for multidisciplinary vigilance and exposes several underexplored areas in the literature.

HIV is a well-established risk factor for persistent infection with oncogenic human papillomavirus (HPV) and the accelerated progression from CIN to invasive cervical carcinoma. Immunosuppression in people living with HIV (PLWH) impairs HPV clearance, even with antiretroviral therapy, contributing to increased rates of high-grade lesions and cervical cancer [1-3]. Studies have shown that HIV-positive women have significantly higher rates of HPV persistence, cervical dysplasia, and cancer progression compared to the general population [1-3]. Despite established screening guidelines, disparities in access to care, delayed colposcopic follow-up, and challenges with patient adherence remain critical obstacles to early detection and treatment [4-7].

Congenital adrenal hyperplasia, most commonly caused by 21-hydroxylase deficiency, results in cortisol deficiency and androgen excess [2,8]. While typically associated with virilization and reproductive complications, its long-term impact on cervical epithelial integrity and cancer susceptibility remains unclear. Some studies have proposed that chronic androgen exposure may alter local immune function, epithelial turnover, or the cervical microbiome, potentially influencing neoplastic risk [8,9]. The anatomical variations in CAH, including abnormal vaginal structure or persistent urogenital sinus, can also complicate examination and delay diagnosis [9]. These anatomical and procedural challenges may necessitate tailored cervical cancer screening strategies for women with CAH, particularly those with surgically reconstructed genitalia. However, the absence of large-scale studies on CAH and gynecologic malignancies represents a significant gap in current understanding.

In addition to routine screening, HPV vaccination should be emphasized in patients with CAH, especially those who are immunocompromised due to chronic glucocorticoid therapy. Early prophylactic immunization may reduce the risk of HPV-related malignancy and should be considered a crucial component of preventative care in this subset.

The development of a vesicovaginal or rectovaginal fistula, as suspected in this case, is a rare but severe complication of invasive cervical cancer, typically arising from direct tumor invasion, infectious destruction, or post-radiation effects [10,11]. In this patient, pneumaturia and air in the vaginal cuff on imaging prompted early suspicion for fistula formation. Fistulas not only impair quality of life but also correlate with poor prognosis and often necessitate surgical intervention [11]. The anatomical and histologic variability of cervical tumors can influence the site and severity of invasion, including the risk of fistula formation [6]. In future reports, inclusion of diagnostic visuals -- such as MRI, vaginoscopy, or colposcopy -- could further enhance the reader’s understanding of disease progression and aid in anatomical correlation.

Atypical symptom presentation and inconsistent follow-up likely contributed to delayed recognition. In immunocompromised patients, symptoms such as abdominal pain, vaginal bleeding, and urinary complaints are often attributed to benign causes, potentially delaying oncologic evaluation. Earlier colposcopic assessment and biopsy may have accelerated diagnosis in this case [4,5].

Importantly, the convergence of HIV and CAH in this patient raises new questions about hormonal and immunological interplay in HPV persistence and cervical carcinogenesis. The combination of immune suppression and altered hormonal environments may create a permissive niche for persistent oncogenic HPV infection and neoplastic transformation [1,2,6,8]. Further studies are needed to evaluate whether CAH-associated endocrine alterations independently modulate the risk of HPV-related malignancies in immunocompromised populations.

Multidisciplinary collaboration between gynecology, infectious disease, endocrinology, and radiology was crucial in this case. Such coordination is essential for improving diagnostic accuracy and patient outcomes in individuals with overlapping comorbidities [10,11].

As with all case reports, the findings presented here represent a single clinical experience and may not be broadly generalizable. However, this case highlights critical clinical considerations that can inform care for patients with similar conditions. The lack of long-term follow-up data due to loss to follow-up is a limitation, and future longitudinal observations would help clarify outcomes and best practices.

## Conclusions

In patients with congenital adrenal hyperplasia and multiple chronic comorbidities, cervical cancer may present atypically due to anatomical alterations and immunological vulnerabilities. Clinicians should maintain a high index of suspicion when evaluating genitourinary symptoms such as pneumaturia, particularly in high-risk individuals. Prompt recognition and early multidisciplinary involvement are essential to achieving optimal outcomes in these complex cases.

HIV infection significantly increases the risk of persistent HPV infection and accelerates progression to cervical cancer, even in patients receiving antiretroviral therapy. In parallel, congenital adrenal hyperplasia (CAH) presents unique gynecologic challenges, yet its role in cervical carcinogenesis remains underexplored. The coexistence of HIV and CAH may further potentiate oncogenic risk through a combination of hormonal imbalances and immune dysregulation. In such immunocompromised patients, atypical symptoms like pneumaturia should raise early suspicion for underlying malignancy or fistula formation, warranting prompt imaging. These complex cases underscore the importance of multidisciplinary collaboration to ensure timely diagnosis and comprehensive management.

## References

[1] Palefsky J (2009). Human papillomavirus-related disease in people with HIV. Curr Opin HIV AIDS.

[2] Speiser PW, Arlt W, Auchus RJ (2018). Congenital adrenal hyperplasia due to steroid 21-hydroxylase deficiency: an Endocrine Society Clinical Practice Guideline. J Clin Endocrinol Metab.

[3] Clifford GM, Franceschi S, Keiser O (2006). Immunodeficiency and the risk of HPV-related and other cancers among persons with HIV/AIDS in the United States. J Natl Cancer Inst.

[4] Massad LS, Xie X, Greenblatt RM (2012). Effect of human immunodeficiency virus infection on the prevalence and incidence of vaginal intraepithelial neoplasia. Obstet Gynecol.

[5] Denny L, Quinn M, Sankaranarayanan R (2006). Screening for cervical cancer in developing countries. Vaccine.

[6] Mahajan NN (2008). Vesicovaginal fistula formation in patients with stage IVA cervical carcinoma. Gynecol Oncol.

[7] Sankaranarayanan R, Nessa A, Esmy PO, Dangou JM (2012). Visual inspection methods for cervical cancer prevention. Best Pract Res Clin Obstet Gynaecol.

[8] Merke DP, Bornstein SR (2005). Congenital adrenal hyperplasia. Lancet.

[9] Chentli F, Belhimer F, Bekkaye I, Azzoug S (2013). Female infertility due to congenital adrenal hyperplasia: diagnosis and treatment. Indian J Endocrinol Metab.

[10] Lalwani S, Varma V, Kumaran V, Mehta N, Nundy S (2015). Complex rectovaginal fistula—an experience at a tertiary care centre. Indian J Surg.

[11] Viswanathan AN, Thomadsen B (2012). American Brachytherapy Society consensus guidelines for locally advanced carcinoma of the cervix. Part I: general principles. Brachytherapy.

